# Treatment of alcohol use disorder in patients with alcohol-associated liver disease: Innovative approaches and a call to action

**DOI:** 10.1186/s13722-024-00448-8

**Published:** 2024-03-19

**Authors:** Lamia Y. Haque, Paola Zuluaga, Robert Muga, Daniel Fuster

**Affiliations:** 1 Department of Medicine, Digestive Diseases, & Program in Addiction Medicine, Yale School of Medicine, New Haven, Connecticut USA; 2 Department of Internal Medicine, Addiction Unit, Hospital Universitari Germans Trias i Pujol, Universitat Autònoma de Barcelona, 08916 Badalona (Barcelona), Spain

**Keywords:** Alcohol use disorder, Alcohol-associated liver disease, Treatment, Addiction medicine, Hepatology

## Abstract

Alcohol-associated liver disease is currently the leading cause of liver transplantation and liver deaths both in Europe and the United States. Efficacious treatments exist for alcohol use disorder, but they are seldomly prescribed for patients who need them. Besides, the presence of liver cirrhosis can complicate pharmacological treatment choices. In this review, we discuss established and innovative treatment strategies to treat unhealthy alcohol use in patients with alcohol-associated liver disease. We also describe the experience of our own institutions, Hospital Universitari Germans Trias i Pujol in Badalona (Spain) and Yale-New Haven Health and Yale Medicine (Connecticut. United States of America).

## Introduction

The prevalence of alcohol use disorder (AUD), the most severe form in the spectrum of unhealthy alcohol use, has increased in recent years [1–3]. Alcohol-associated liver disease (ALD) is one of the most common alcohol-related medical problems and is responsible for a sizable number of liver-related deaths [4, 5]. Deaths due to alcohol-associated cirrhosis and alcohol-related liver cancer account for 1% of all deaths worldwide, but this figure may be an underestimation [6].

Although efficacious treatments exist for AUD [7], they are seldomly prescribed in reality, especially among patients with end stage liver disease [8, 9]. Therefore, there is an urgent need for innovative treatment strategies to improve care for this often marginalized subset of patients [10].

Recent guidelines on the management of ALD have highlighted the need for better communication and collaboration between addiction medicine specialists and hepatology specialists [11]. In the present review we aim to summarize innovative approaches for the treatment of AUD in patients with ALD. We will also describe our experience in our institutions (Yale School of Medicine in Connecticut, U.S. and Hospital Universitari Germans Trias i Pujol in Badalona, Spain).

### Epidemiology of ALD

ALD is one of the most common causes of chronic liver disease and is presently the leading indication for liver transplantation both in Europe and the United States [11–15]. ALD ranges in severity from steatosis to acute alcohol-associated hepatitis and cirrhosis [12]. Deaths due to cirrhosis in general have been rising over the past decade, largely driven by the rise in ALD [4, 16, 17]. Notably, there has been a disproportionate increase in morbidity and mortality due to ALD among younger adults, women, and minoritized groups [4]. Individuals with cirrhosis due to ALD are more likely to present at more advanced stages of disease, including portal hypertensive complications, and have higher rates of hospitalizations compared with other etiologies of liver disease [18]. Furthermore, the burden of ALD has expanded in the context of the coronavirus disease 2019 (COVID-19) pandemic, with worsening mortality and higher rates of liver transplantation, attributed to the increase in alcohol use that has occurred. A recent study performed in the United States has shown that ALD mortality has increased from 2017 to 2020 and has accelerated further during the COVID-19 pandemic [19]. Specifically, age-adjusted ALD-related mortality rates per 100,000 increased each year from 2017 through 2020 for both sexes; mortality rates rose from 13.1 (95% confidence interval CI 12.9–13.3) in 2017 to 16.9 (95% CI 16.7–17.1) in 2020 among males and from 5.6 (95% CI 5.4–5.7) in 2017 to 7.7 (95% CI 7.6–7.9) in 2020 among females [19].

### Assessment of unhealthy alcohol use

The National Institute on Alcohol Abuse and Alcoholism (NIAAA) recommends that all patients be assessed for unhealthy alcohol use during all routine clinical visits, especially those who have potentially alcohol-related health problems like liver disease [20]. The NIAAA Single Alcohol Screening Question could be a first step, and is as follows: “How many times in the past year have you had four (for women or anyone age 65 or older)/five (for men under the age of 65) or more drinks in a day?,” where X is five for men and four for women, and a response of one or more times is considered positive [21].

All positive screenings should be followed by the AUDIT-C or another validated measure to characterize alcohol use. AUDIT-C is a short version of the full AUDIT alcohol questionnaire and includes 3 questions of the AUDIT. A score ≥ 3 for women and ≥ 4 for men is considered positive for unhealthy alcohol use [22]. The full AUDIT questionnaire consists of 10 questions; and a score 1–7 is consistent with low-risk alcohol consumption, while a score between 8 and 14 is suggestive of hazardous or harmful alcohol consumption, and a score of 15 or more has a high likelihood of what was defined as alcohol dependence and is now defined as moderate-severe AUD [23]. For the diagnosis of AUD, physicians should use DSM-5, which includes 11 different diagnostic criteria that take into account absence of control, social consequences, risks associated with alcohol use as well as tolerance and dependence. AUD can be classified as mild (2–3 criteria), moderate (4–5 criteria) or severe (≥ 6 criteria) [24].

### Treatment of AUD and Outcomes in ALD

The cornerstone of treatment for ALD at any stage is cessation of alcohol intake [11]. Abstinence from alcohol is the most significant predictor of survival among patients ALD [11, 25, 26]_._ Among patients who experience an episode of acute alcohol-associated hepatitis, even the lowest levels of alcohol intake are associated with greater mortality; furthermore, mortality rises in a dose-dependent fashion correlating with the quantity of alcohol consumed with hazard ratios of 2.36, 3.2, 3.51, and 5.61 for 1–29 g per day, 30–49 g per day, 50–99 g per day, and over 100 g per day, respectively, as described in a prospective cohort study [25]. Despite the significant harms associated with alcohol consumption in ALD, alcohol use remains common among patients with this condition [25–27]. Among patients who survive an episode of acute alcohol-associated hepatitis, the risk of self-reported alcohol use at one and five years is 25.2% and 35.2%, respectively [25]. Based on a single-center study of patients with alcohol-associated hepatitis and cirrhosis, 50% report having alcohol within the past year [27]. Finally, alcohol use is also common after liver transplantation, with up to 34% of patients reporting alcohol consumption within two years and with associated decreases in long-term survival [15, 28, 29].

Treatment of AUD can have significant implications for liver-related health in patients with ALD. In a cohort study of over 35,000 patients with cirrhosis due to ALD, AUD treatment is associated with lower rates of hepatic decompensation [adjusted odds ratio 0.63; 95% confidence interval (0.52, 0.76)] as well as decreased long-term mortality [adjusted odds ratio 0.87; 95% confidence interval (0.80, 0.96)] [30]. A second observational study of over 1000 patients with ALD also supports the association between AUD treatment and a reduction in hepatic decompensation, and additionally shows that patients who receive medications for AUD prior to the development of clinically apparent ALD are also less likely to develop incident ALD [adjusted odds ratio 0.37; 95% confidence interval (0.31–0.43)] [31]. In addition, patients who receive behavioral treatment for AUD, such as individual and group psychotherapy, are also less likely to develop incident ALD [hazard ratio 0.59; 95% confidence interval (0.50–0.71)] or hepatic decompensation for those with pre-existing ALD [hazard ratio 0.86; 95% confidence interval (0.48–0.95)] [32]. These findings highlight the importance of linking patients with ALD to AUD treatment and, indeed, society guidelines and consensus statements on the management of ALD recommend that clinicians caring for patients with ALD provide or facilitate AUD treatment [10, 11].

### Patient identification and early detection of ALD in AUD

Early detection of ALD among patients with unhealthy alcohol use is of utmost importance given that, as previously mentioned, ALD is often diagnosed once the disease is already advanced [18]. Almost three quarters of patients with ALD present with a non-elective hospital admission because of decompensated cirrhosis [33, 34]. The presence of decompensated cirrhosis may limit treatment options for AUD, as patients who are medically complex may be unable to participate in intensive behavioral treatments, and certain pharmacotherapy options may no longer be safe [8]. In addition, advanced ALD can also complicate the management of alcohol withdrawal, which mainly consists of benzodiazepines, and requires judicious dosing and careful monitoring [8, 9].

Targeting high risk populations is among the strategies described in a recent paper outlining a blueprint for action to tackle ALD [10]. Identifying and staging the severity of ALD among patients with AUD can facilitate early intervention and prevent future complications, as alcohol reduction and cessation at any stage of ALD is associated with benefits. Furthermore, holistic care that includes nutritional support and modification of additional risk factors such as overweight or obesity alongside treatment of AUD may provide further benefit. An optimal nutritional assessment with recommendations that support weight loss if indicated can reduce the risk of additional liver injury from metabolic dysfunction-associated steatohepatitis in patients with unhealthy alcohol use who are obese or overweight [35–37].

### Current treatment options for AUD in patients with ALD

Treatment options for unhealthy alcohol use and AUD in ALD have been discussed extensively in several review articles in recent years [1, 8, 9, 11, 12, 38–41].

#### Non-pharmacological treatment

Brief intervention and motivational interviewing have been associated with reductions in drinking in the setting of unhealthy alcohol use that does not meet the criteria for AUD [42, 43]. In addition to brief interventions, feedback around abnormal laboratory tests is associated with decreased alcohol intake among patients at risk for progressive liver disease [44].

Patients with more severe cases of unhealthy alcohol use, including AUD, should be offered more intensive forms of treatment. Among non-pharmacologic treatments, cognitive behavioral therapy and motivational enhancement therapy have the most robust evidence [45]. In a systematic review specifically focused on behavioral treatments such as psycho-education, contingency management, cognitive behavioral therapy, motivational interviewing, and motivational enhancement therapy, only one randomized controlled trial that identified a statistically significant increase in abstinence through these interventions was included, and this trial reported higher levels of treatment engagement than other studies [45]. Observational studies have also demonstrated an association between behavioral interventions and abstinence [45].

Furthermore, providing behavioral treatment integrated with liver disease management have been shown to improve rates of abstinence. Three observational studies and one randomized controlled trial utilizing this approach, in which liver disease care and behavioral interventions were provided through a singular provider or through a multidisciplinary team with close communication, demonstrated a positive impact on rates of abstinence regardless of the severity of liver disease, and this effect was more robust than that seen in studies in which care was not integrated [45]. Of the five observational and four randomized controlled trials evaluating behavioral treatment for ALD in a non-integrated fashion, there was a positive correlation between behavioral treatment and abstinence, however the impact appeared lower that of integrated therapy [45]. Of note, although no behavioral intervention was shown to be associated with long-term abstinence, an integrated approach using cognitive behavioral therapy combined with medical care reduced alcohol consumption [45].

In a study that included 105 male patients with severe medical complications caused by unhealthy alcohol use and recent drinking, an integrated outpatient treatment program combining cognitive behavioral therapy and motivational enhancement therapy delivered over two years led to abstinence (74% vs. 47%; p = 0.02) [46]. Standard medical care alone was also surprisingly effective in promoting abstinence in this patient population. Integrated outpatient treatment significantly increased both treatment engagement and abstinence for a modest annual cost [46].

In another study that combined medical care with cognitive behavioral therapy, significantly lower rates of patients with alcohol-associated cirrhosis who underwent liver transplantation returned to alcohol use after receiving this care (32.7% vs. 75%, p = 0.03) [47]. Although these findings are compelling, it is possible that patients who undergo liver transplantation are a selected group that may not be representative of the broader population of patients with ALD [47]. All in all, behavioral treatments that are delivered in models that integrate care result in better outcomes than approaches which are not integrated.

#### Pharmacological treatment

Treating AUD involves the use of medications to treat alcohol withdrawal as well as medications that promote abstinence primarily by reducing alcohol cravings.

##### Alcohol withdrawal treatment

Oral benzodiazepines are the cornerstone of treatment for alcohol withdrawal syndrome. Generally, a symptom-driven approach to benzodiazepine administration is recommended using standardized assessments [8]. In patients with preserved liver function, diazepam or clordiazepoxide are often used, however in patients with advanced liver disease, lorazepam or oxazepam are preferred, as they are not hepatically metabolized. Sodium oxybate (the sodium salt of gamma-hidroxibutiric acid) is approved for the treatment of alcohol withdrawal in Italy and Austria, and may be considered as a second-line treatment when other established drugs are deemed as not appropriate [48, 49]. Information around the use of other drugs is scarce, and limited experience exits with the use of antipsychotics [8].

##### Treatment to promote abstinence and prevent relapse

Disulfiram, naltrexone, and acamprosate are approved by the United States Food and Drug Administration (FDA) and the European Medicines Agency to treat AUD [8]. Nalmefene is approved in Europe to reduce heavy drinking and its use is recommended as a harm reduction strategy for patients with heavy episodic drinking that do not meet criteria for AUD [8]. Overall, despite their efficacy, only 10% of those with AUD receive any form of treatment, and less than 1% of those with AUD receive any FDA-approved medications [50, 51].

Disulfiram inhibits acetaldehyde dehydrogenase and provokes an acetaldehyde syndrome when consumed with alcohol. Its efficacy is limited by adherence and appears most effective among highly motivated patients or in directly monitored settings. Significant concerns around liver toxicity exist for patients with advanced liver disease and cirrhosis, and it is not recommended to be used in patients with ALD [38, 52] due to rare instances of fulminant liver injury and death in these settings [11, 39, 53]. Therefore, ruling out the presence of cirrhosis is recommended before disulfiram is started [11, 54].

Naltrexone is an opioid receptor antagonist that decreases cravings to drink and is available in oral and injectable extended-release formulations [8]. The number needed to treat for naltrexone is 12 for reductions in heavy drinking and 20 for complete abstinence [55–57]. Naltrexone is primarily contraindicated in patients with concurrent opioid use [8]. Naltrexone previously had a “black box warning” due to association with hepatotoxicity, however this has since been removed for several reasons [58, 59]. First, the doses of naltrexone that are associated with hepatotoxicity (> 300 mg) far exceed the approved dose for the treatment of AUD (50 mg). Furthermore, there are no reports of liver failure due to naltrexone, as has been the case with disulfiram. In fact, a recent retrospective study describing the experience of 160 patients who received naltrexone in a single center in the United States, including patients with both compensated and decompensated cirrhosis, showed that there were very few cases of liver enzyme elevation [60]. Among the two patients in whom liver injury occurred and did not resolve after naltrexone discontinuation, a more likely explanation was liver injury due to alcohol use [60]. These findings further support the idea that aside from patients with progressive liver failure in whom naltrexone metabolism may be significantly impaired, the benefits of naltrexone may outweigh the uncommon risk of liver injury in most patients with ALD.

Nalmefene is another opioid receptor antagonist that is not associated with significant liver toxicity, but caution may be exercised as its clearance diminished in end-stage liver disease [61]. Nalmefene is approved in Europe for the reduction of alcohol use among those with unhealthy alcohol use [8]. Because abstinence rather than reduction in drinking is often the treatment goal for patients with ALD, use of nalmefene may be less relevant in this setting [8, 61].

Acamprosate, which modulates NMDA receptor signaling, is associated with a reduction of alcohol consumption as well as abstinence in AUD. There have not been reports of hepatotoxicity and is felt to be safe in patients with ALD without significant renal impairment [62, 63]. The number needed to treat is 9 for reductions in heavy drinking. Adherence is the primary challenge with this medication due to the frequency of dosing and pill burden. Caution is advised in patients with impaired renal function, as its use is contraindicated with creatinine clearances below 30 ml per minute [8].

Several off-label medications have had growing evidence to support their use in AUD and can be considered as options in patients with ALD. Although further evidence in patients with advanced liver disease is needed, several off-label medications, including baclofen and topiramate, are included in guidelines for the management of ALD by the American Association for the Study of Liver Diseases and the European Association for the Study of the Liver [11, 64]. Baclofen, a GABA-B receptor agonist, is frequently used as an off-label medication for the treatment of AUD in France [65, 66], and has been tested in several trials, mainly performed in Italy, among patients with cirrhosis showing efficacy in promoting abstinence [9]. Additional data have been mixed, including more recent studies that did not show improvements in alcohol use among patients with AUD receiving baclofen [67]. Trials comparing baclofen to other medications such as naltrexone or acamprosate are lacking. In addition, there may be harm associated with baclofen at high doses [68], baclofen may cause mental status changes and exacerbate encephalopathy [12, 38]. Despite this, baclofen is used relatively more commonly among patients with ALD and AUD, especially in patients with persistent cravings for alcohol [9]. Notably, French guidelines suggest that doses not exceed 80 mg per day [9].

Topiramate, an antiepileptic with multiple effects including enhancement of GABA-A receptor activity and blockage of voltage-gated calcium channels, is also considered as a possible option in patients with ALD and may have additional benefits for those with co-morbid migraine headaches, obesity, or binge eating disorders [12]. It should also be used with caution in patients with hepatic encephalopathy, as some of its side effects including alterations in cognition can confound symptoms of encephalopathy [8, 12].

Gabapentin, another anticonvulsant that modulates calcium channel signaling, has been used in the treatment of alcohol withdrawal symptoms and for AUD. It can be considered in patients with peripheral neuropathy, restless leg syndrome, insomnia or anxiety. It can also be used both to prevent withdrawal and reduce cravings. The evidence supporting the use of gabapentin as a second-line treatment for preventing alcohol withdrawal syndrome in selected patients also exists in addition to its role in treating AUD [40, 69].

The favorable numbers needed to treat for both naltrexone and acamprosate suggest that the effectiveness of these treatments is on par with the treatment for many other chronic conditions. In addition, a recent meta-analysis indicated that a range of different medications including off-label options, may be as effective as those that are FDA-approved [70]. Furthermore, receipt of medications for AUD as well as other treatments in those with ALD has been associated with improvements in liver-related outcomes. Although seldom prescribed for patients ALD, these treatments along with counseling may even be cost-effective [71]. A retrospective study of a large cohort of over 30,000 patients with cirrhosis [30] found that any AUD treatment was associated with a significant reduction in hepatic decompensation [adjusted odds ratio 0.63 (95% CI 0.52, 0.76)] and in long-term all-cause mortality [AOR 0.87 (95% CI 0.80, 0.96)]. A more recent retrospective study of over 9000 patients with AUD among whom 11.8% had ALD at baseline demonstrated that medications for AUD were associated with a lower incidence of ALD and a lower incidence of hepatic decompensation in those who already had ALD [31]. Associations were also found for individual therapies, such as for naltrexone, gabapentin, topiramate and baclofen for reduced incidence of ALD; and for naltrexone and gabapentin for lower incidence of hepatic decompensation. The use of acamprosate was associated with an increased risk of ALD and a trend toward greater decompensation, potentially due to bias by indication, as patients with clinically apparent liver disease may be more likely to receive this medication due to its lack of association with liver injury [31].

### The AUD treatment gap in ALD

Despite the strong association between alcohol intake and mortality in ALD as well as emerging evidence demonstrating the beneficial impact of AUD treatment on clinical outcomes in those with ALD, few individuals with ALD receive AUD care [27, 30, 50, 72]. In an observational study of patients who were recently hospitalized with complications of cirrhosis with co-occurring AUD as well as other substance use disorders, only 35% report establishing AUD treatment when presenting for outpatient hepatology follow up care [72]. Similarly, in a large cohort of patients with cirrhosis due to ALD and AUD receiving care at the Veterans Health Administration in the United States, where mental health and addiction treatment are available for all enrollees, only 14% received any treatment for AUD within six months of receiving an AUD diagnosis [30]. Furthermore, less than 2% of patients with ALD and AUD in this cohort received medications for AUD [30]. In another study of over 60,000 privately-insured patients with cirrhosis due to ALD in the United States, 10% engaged in visits with mental health or addiction treatment clinicians and less than 1% received medications for AUD [50]. Of note, women were less likely than men to receive care for AUD compared with men, both in the form of visits with clinicians and in terms of medications for AUD, with hazard ratios of 0.84 and 0.89, respectively [50]. Efforts to decrease gender-specific barriers are needed, especially considering that women face greater alcohol-associated stigma than men. Also, women develop ALD-related complications after exposure to smaller amounts of alcohol compared with men [73].

Several patient, clinician, and system-level barriers to AUD treatment among patients with ALD have been described [27, 74–77]. Patients with ALD report factors such as limited social support, transportation, insurance coverage, or financial resources as barriers to AUD treatment [27, 74]. Patients may also have limited understanding or insight regarding AUD, perceive a lack of benefit from AUD treatment, and be deterred by stigma associated with AUD treatment [27, 74]. Clinicians who care for patients with ALD commonly report feeling unprepared to deliver or facilitate AUD treatment [76, 77]. According to a nationally representative survey of over 400 hepatology clinicians in the United States, 71% never prescribed any medications for AUD and 90% felt they needed more training regarding the management of AUD [76]. Finally, system-level factors including shortages among addiction specialists, limited AUD treatment resources, separation between ALD and AUD treatment teams, as well as inequities in access to AUD care affect patients’ ability to receive AUD treatment [74, 78]. The low levels of AUD treatment receipt among patients with ALD highlight the pressing need for new approaches to transcend these barriers and underscore the importance of emerging integrated care models for ALD and AUD.

### Integrated care models for AUD and ALD

There is wide variation in the implementation of non-pharmacological and pharmacological interventions for AUD.

Among the most innovative approaches to treating AUD among patients with ALD is integrated care. Integrated care models for ALD and AUD aim to facilitate or directly provide treatment for both ALD and AUD simultaneously [74, 78]. Care models for ALD and AUD can range from “unaffiliated,” in which clinicians who treat ALD and AUD provide care in separate locations without any collaboration, to “co-located,” in which clinicians who treat ALD and AUD treat patients side by side in the same clinical setting [78]. Integrated care models are often multidisciplinary, inter-professional, and team-based, consisting of clinicians with training in hepatology, addiction medicine, psychiatry, psychology, social work, and nursing, and often require the support of clinical leadership, shifts in organizational culture, and new approaches to patient care [78].

Several forms of integrated care for ALD and AUD are described in hepatology care settings. An early example involving a 24-week single-arm prospective behavioral intervention consisting of individual and group counseling integrated within a hepatology clinic for patients with hepatitis C virus (HCV) infection and unhealthy alcohol use based on Alcohol Use Disorders Identification Test screening results shows feasibility of this model as well as reductions in the Addiction Severity Index [79]. A subsequent multicenter pragmatic randomized trial by the same group in which screening, brief intervention, and referral to treatment alone (SBIRT) is compared with SBIRT plus co-located integrated behavioral treatment for AUD for patients with HCV indicates a decrease in alcohol consumption in terms of grams of alcohol consumed as well as heavy drinking days and an increase in the proportion of patients with abstinence from alcohol in both treatment arms without significant differences between them [80]. Behavioral treatment for AUD including evidence-based strategies such as cognitive behavioral therapy as well as motivational enhancement therapy delivered either in-person or via telephone encounters, were feasible in this care setting [80]. In addition to care models incorporating components of addiction treatment into existing hepatology clinics, comprehensive multidisciplinary programs integrating treatment of ALD and AUD in the outpatient setting are also emerging [81, 82]. One clinic involving a hepatologist, psychiatrist, psychologist, nurse, and social worker caring together for patients with advanced ALD including cirrhosis and acute alcohol-associated hepatitis shows the feasibility of this robust inter-professional model as well as improvements in outcomes such as hospital utilization after engagement in care [81]. Notably, patients in this clinic meet all members of the multidisciplinary team during an initial half-day clinic visit and are contacted by the clinic team prior to their first visit to encourage and confirm attendance [81]. Patients receive educational materials and have a comprehensive assessment of their liver health, alcohol and other substance use, as well as psychiatric symptoms [81]. All clinicians in the multidisciplinary team are trained in motivational interviewing and at the conclusion of each half-day session, the team meets to determine an individualized treatment plan for each patient for both ALD and AUD, the latter consisting of outpatient behavioral and pharmacologic interventions as well as referrals to higher levels of care if warranted [81].

Inpatient hospitalizations are frequent among patients with ALD and AUD and are an additional opportunity for engagement in integrated care [83]. In one pilot study, a dedicated inpatient team consisting of a hepatologist and nurse practitioner provide liver-focused evaluations for patients with AUD who are hospitalized with conditions such as alcohol withdrawal but may not have active liver-related concerns [84]. Patients are offered non-invasive liver fibrosis testing and follow up care in an outpatient ALD clinic, and are often seen in parallel by an inpatient addiction medicine consult service [84]. This model of care is associated with greater establishment of outpatient hepatology care for patients earlier in the ALD disease trajectory [84].

Integrated care models for ALD and AUD in the context of liver transplantation are associated with decreased rates of alcohol consumption both before and after transplantation [47, 85–87]. In one of the earliest such models, an Alcohol Addiction Unit embedded within a liver transplantation program in Italy, has been associated with reductions in self-reported alcohol intake as well as mortality [47]. A more recent French study indicated that among patients cared for by addiction specialists within the liver transplant center harmful relapse occurs in less than 10% [85]. Most recently, a pilot program providing integrated ALD and AUD care in Canada with the goal of facilitating liver transplantation for patients with less than six months of abstinence from alcohol has also demonstrated feasibility [86]. Consisting of a multidisciplinary team including hepatologists, addiction and consultation-liaison psychiatrists, social workers, and a nurse practitioner, this integrated model highlights that holistic, multidisciplinary care including treatment for AUD as well as alcohol biomarker monitoring is associated with low levels of alcohol use and robust survival after transplantation [86]. As the burden of severe ALD and consequent need for liver transplantation for this indication rises, the incorporation of AUD treatment in the care of patients with advanced ALD before and after transplantation is of growing importance [88].

### Our experiences in implementing integrated care for alcohol use disorder and alcohol-associated liver disease

#### Hospital universitari Germans Trias i Pujol Badalona, Spain

In Hospital Universitari Germans Trias i Pujol, an Addiction Medicine consult has been performed for all patients with ALD admitted at the Hepatology Department since 2015. Physicians from the Addiction Unit within the Internal Medicine Department assess all patients with ALD to rule out alcohol withdrawal, to evaluate alcohol consumption, and to prescribe medications, either to treat alcohol withdrawal or to prevent relapse in alcohol use, as deemed appropriate.

In addition, a brief intervention is performed to stress the importance of abstinence from alcohol consumption in the clinical course of ALD. All patients are followed during admission and are then referred to the Addiction Medicine outpatient clinic upon discharge. They are then followed every 8 weeks until a 6-month abstinence period is achieved.

In the period between years 2016 and early 2022, 209 consecutive consults have been performed in 165 different patients (81.2% male, median age 55 years) with unhealthy alcohol use and ALD admitted to the Hepatology Department. Of note, one patient has been evaluated in 5 different admissions, 2 patients in 4 different admissions, 10 patients in 3 different admissions, and 13 in two different admissions. The remaining 139 patients have been evaluated in a single admission.

The reason for admission was decompensation of cirrhosis of the liver in 114 admissions, most commonly because of ascites (64 admissions) or variceal bleeding (30 admissions). Acute alcohol-associated hepatitis was responsible for 59 admissions. Of interest, 30 patients presented with alcohol withdrawal that required an increase in the initial dosage of medications prescribed by the hepatologists. The majority of patients that could be safely discharged did attend the Addiction Medicine Outpatient clinic.

Other subsets of patients with ALD that are followed in the Addiction Medicine outpatient clinic include patients in the liver transplant waiting list, patients with unhealthy alcohol use and other forms of liver disease and patients with less advanced forms of ALD, as well as patients who have been admitted to the hospital for alcohol detoxification and in whom ALD has been detected.

In fact, in the series of patients with alcohol use disorder who are admitted for hospital treatment of the disorder, subclinical alcohol-liver disease is not uncommon. We have reported that only 23% of patients had a totally normal liver ultrasound [89]. For example, 25% have a Fibrosis-4 Index for Liver Fibrosis (FIB-4) value greater than 3.25, which is consistent with advanced liver fibrosis [89]. In our experience, survival of patients with FIB-4 values suggestive of advanced liver fibrosis or with alcohol-associated liver injury, defined as the presence of two or more of the following criteria: AST elevation between 74 and 300 U/L, AST/ALT ≥ 2 and/ or total bilirubin > 1.2 mg/dL, is poorer than for patients with AUD and normal liver enzyme tests [90, 91].

#### Yale-New Haven health and Yale medicine (CT), USA

Patients with ALD and AUD at Yale-New Haven Health and Yale Medicine receive care through integrated and collaborative models. The Yale Clinic for Alcohol and Addiction Treatment in Hepatology, led by a clinician with dual training in hepatology and addiction medicine, is embedded within a large outpatient academic hepatology practice affiliated with a tertiary care center [92]. Patients with ALD and AUD are referred from primary care, gastroenterology, and hepatology practices in the community and within the health care system as well as from inpatient hepatology teams caring for hospitalized patients with ALD and AUD for outpatient care after discharge. Patients are offered onsite integrated treatment for ALD, AUD, and other substance use disorders, including medications for AUD, as well as linkage to behavioral health and peer support services. While hospitalized, patients with ALD and AUD are often cared for by both the inpatient hepatology primary or consult services as well as the Yale Addiction Medicine Consult Service, allowing patients to receive comprehensive treatment for both conditions simultaneously [92, 93]. Integration of ALD and AUD care at Yale is supported by the Yale Program in Addiction Medicine, which has a longstanding history of integrating treatment of AUD and other substance use disorders into a range of medical settings including primary care, emergency departments, and subspecialty clinics [94–100].

**Fig. 1:**
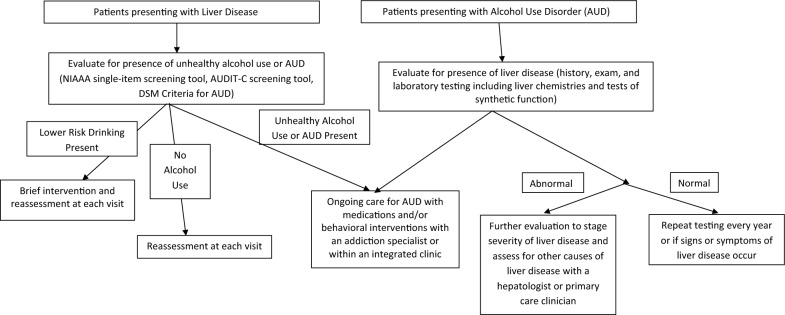
Goals for integrated and collaborative care for patients with AUD and ALD. Treating AUD and promoting abstinence is the primary goal among patients with unhealthy alcohol use of AUD to prevent evolution to end stage liver disease

Figure 1 shows our proposed approach for patients with unhealthy alcohol use and liver disease.

## Conclusions

Multiple approaches have been described to better treat AUD among patients with ALD. Despite that, there is a huge treatment gap and there is a large population that would benefit from integration of addiction medicine and hepatology care. To reduce the global burden of ALD, streamlined pathways facilitating linkage to treatment as well as innovative multidisciplinary clinics should be adopted as the current treatment paradigm [10]. In addition, improved education for hepatologists on the treatment of AUD as well as methods of collaborating with addiction medicine specialists and building integrated models are needed [39, 101].

## Data Availability

Not applicable.
